# Migrant and minority health in Europe: the way forward: Report on the Salzburg Workshop on Migrant and Minority Health, 3–9 April 2016

**DOI:** 10.1186/s40985-016-0045-0

**Published:** 2016-11-28

**Authors:** Tina Bregant, Mariam Torosyan, Amanda Shriwise, Lukasz Balwicki, Ted Tulchinsky

**Affiliations:** 1 University Rehabilitation Institute, Republic of Slovenia, Ljubljana, Slovenia; 2 Faculty of Public Health, Yerevan State Medical University, Yerevan, Armenia; 3 Institute of Philosophy, Sociology and Law, National Academy of Sciences of the Republic of Armenia, Yerevan, Armenia; 4 Department of Sociology, Harvard University, Cambridge, MA USA; 5 Department of Social Policy and Intervention, University of Oxford, Oxford, UK; 6 Department of Public Health and Social Medicine, Medical University of Gdansk, Gdansk, Poland; 7 Braun School of Public Health and Community Medicine, Hebrew University Hadassah, Jerusalem, Israel; 8 School of Health Sciences, Ashkelon Academic College, Ashkelon, Israel

**Keywords:** Migrant health, Minority health, Europe, Public health

## Abstract

Migrant and minority health has always been an issue of special concern in public health. While migration is not a new phenomenon, the number of refugees and migrants across the globe grew rapidly in 2015, with large numbers from the Middle East and Africa. Furthermore, the recent migrant crisis in Europe—sparked by civil wars in Syria and Libya and continuing conflict in Iraq and Afghanistan—has escalated to the level of a humanitarian emergency requiring immediate action.

We conducted an international workshop on migrant and minority health in Salzburg from 3 to 9 April 2016 to examine migrant and minority health issues in greater depth, sponsored by the American Austrian Foundation and in cooperation with the Association of Schools of Public Health in the European Region (ASPHER) and *Public Health Reviews* (*PHR*). To continue this discussion within the academic literature, *PHR*’s special issue on migrant and minority health includes articles from conference participants and other experts in medicine and public health from the European region and beyond.

Informed by the contribution of senior representatives of the European Union, the International Organization for Migration (IOM), Médecins Sans Frontières (MSF), and public health practitioners and investigators from over 30 countries, this editorial summarizes recommendations of the conference participants for improving migrant and minority health in Europe. They include (i) developing a conceptual framework for health care intervention for migrants, (ii) oversight and coordination of migrant and minority health activities, (iii) reaching a consensus on implementation practices, and (iv) mobilizing sufficient resources for addressing the health needs of migrants.

## Introduction

The American Austrian Foundation and *PHR*-ASPHER conducted an international workshop on migrant and minority health in Salzburg from 3 to 9 April 2016.^1^ *Public Health Reviews* (*PHR*) is now publishing a special issue on migrant and minority health, and the conference participants included both authors of articles for this special issue and representatives of the Association of Schools of Public Health in the European Region (ASPHER) member schools across the European region.

Migrant and minority health has always been an issue of special concern in public health. The recent migrant crisis in Europe—sparked by civil wars in Syria and Libya and continuing conflict in Iraq and Afghanistan—has escalated to the level of a humanitarian emergency requiring immediate action [1, 2]. While migration is not a new phenomenon, the number of refugees and migrants has grown rapidly in the past year, with large numbers originating from the Middle East and Africa. As of January 2015, the Office of the United Nations High Commissioner for Refugees (UNHCR) counts a total of 13,685,607 refugees worldwide and lists the total population of concern at 54,945,467 [3]. In Europe, UNHCR and the International Organization for Migration (IOM) estimate that over one million men, women, and children arrived in 2015 [4]; nearly 190,000 additional migrants have arrived as of May 2016, with many more continuing to make the arduous journey [5].

To examine migrant and minority health issues in greater depth, the Salzburg Workshop included presentations from senior representatives of the European Union, IOM, Médecins Sans Frontières (MSF), as well as public health practitioners and investigators from over 30 countries in Europe and beyond. Presentations were given on international organizations and also on national experiences with migrant and minority health issues. Workshop participants contributed to one of three working groups examining (i) the political aspects of the crisis, (ii) the public health effects, and (iii) their social implications.

## Principles

From the outset, the Workshop participants recognized the following internationally sanctioned UN declarations and conventions as the foundation for understanding the current migration crisis: the 1948 Universal Declaration of Human Rights [6], the 1948 Convention on Prevention and Punishment for the Crime of Genocide [7], and the 1951 Convention Relating to the Status of Refugees—including the 1967 Protocol Relating to the Status of Refugees and Resolution 2198 (XXI) [8]. These documents establish the international norms of human rights, protection from genocide, and the rights of refugees. Despite these standards—many of which emerged in response to the bitter and tragic experience of World War II and the Holocaust, including the refugee crisis in its aftermath—genocide and forced migration continue. The current migration crisis in Europe has led to a renewed global commitment and a re-doubling of efforts to substantiate the rights of individuals seeking refuge from conflicts in the Middle East, Africa, the Balkans, and other parts of the world.

## Key issues

**Fig. 1:**
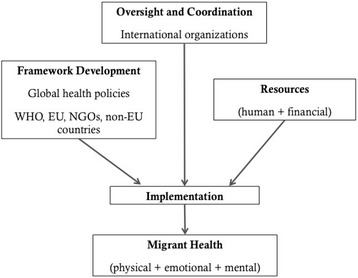
Coordinating migrant health policy

Four key issues were identified to address migrant and minority health: (1) development of a conceptual framework for health care intervention, (2) oversight and coordination of migrant and minority health activities, (3) consensus on implementation practices, and (4) mobilization of sufficient resources. Figure 1 illustrates how attention to these key issues contributes to the coordination of migrant health policy.

A conceptual framework for addressing migrant health care interventions must consider and harmonize both health care and public health policies and practices. International organizations have a fundamental role to play in the oversight and coordination of migrant and minority health. Effective leadership from international organizations is critical, particularly for monitoring health and for coordinating health responses across borders. Achieving a consensus on implementation practices, such as a standard set of international health guidelines, could better facilitate communication between health professionals and ensure that theoretical principles are translated into clinical practice in a way that is both consistent and evidence-based. Finally, sufficient resources must be dedicated to providing public health protection and medical care to those in need. This includes educating policy makers, supervisory and health service staff, and community health workers on the unique health needs of migrants and minorities, which can be informed and facilitated by health care professionals with international experience.

Migrant and minority health includes not only physical health but also mental and emotional health. While the physical and material needs of refugees—shelter, safety, warmth, nutrition, sanitation, and health care—often take priority, the mental and emotional health needs of migrants are just as integral to their well-being. This includes encouraging self-actualization, supporting avenues for self-help, providing opportunities for gainful employment, and fostering a sense of belonging and self-esteem [9]. Help in adapting to a new cultural environment and lifestyle, such as learning the local language, norms, and customs, is vital to successful assimilation and integration of migrants and minorities in a way that preserves their dignity, independence, and self-respect as well as commitment to absorption and inclusion in their host country.

## Discussion and recommendations

Facilitated by the three working groups, the Workshop participants prepared a summary and draft recommendations related to the political, social, and public health aspects of the migrant crisis. Problem areas identified in migrant and minority health, along with examples and possible interventions, are listed in Table 1.

**Table 1:** Problem areas and possible interventions to improve migrant and minority health

Problem area	Specific problem	Example policy intervention
Knowledge gap in migrant health	Policymakers, supervisory staff, and direct care givers lack training on migrant health issues. Community health workers are not adequately prepared to address the unique needs of refugees and their families.	Provide training to public health leaders, including policymakers, supervisory staff, direct care workers, and community health workers Consult migrants and refugees themselves about how best to sustain and promote migrant health
Basic needs provision	Inadequate and/or deteriorating shelter, sanitation, waste disposal, and clean water facilities	Increase monitoring of water supply and sanitary facilities Increase repair and supply of facilities as needed and feed this information back into plans for preparedness and response
Nutritional security	Inadequate caloric intake of healthy protein, carbohydrates and fats with natural vitamins, minerals, and antioxidants	Ensure provision of vitamin- and mineral-fortified foods Distribute vitamin and mineral supplements, particularly for the most vulnerable migrants including children, pregnant women, and the elderly
Infectious disease control	Changes in the risk, prevalence, and incidence portfolios of infectious diseases, including Hepatitis (A, B, C), HIV/AIDS, malaria, tuberculosis, poliomyelitis, influenza, and other sexually transmitted diseases	Promote screening, vaccination, and treatment among migrant populations Facilitate better health record keeping for migrants Harmonize vaccination policy across the European Region
Mental health provision	Increase in depression, anxiety, post-traumatic stress disorder (PTSD) among migrant populations	Access to use of mental health services Education, self-help group activities, surveys, and specific interventions
Access to medical care	Emergency care Lack of access to regular medical care, often resulting in an increase in demand for emergency services	Improve migrant access to the health system Curative and preventive medicine interventions
Non-communicable disease	Increased vulnerability to acquiring non-communicable diseases as a result of the migrant journey	Promote increased education and preventive screenings Improve health monitoring of non-communicable diseases among migrants, particularly through improved health record keeping (both e-records and hand carried printed records)
Maternal and child health	Managing changes in risk for nutritional disorders (including problems with breastfeeding), exposure to violence and trafficking, and other factors affecting women’s sexual and reproductive health	Promote micronutrient-fortified (e.g., vitamins A, B, C, D, iodine, iron, folic acid) food staples (e.g., flour, milk, salt) Enhance protection against female genital mutilation, sexual exploitation, and child marriages at all stages of the migrant journey Promote migrant and refugee education programs focused on adapting to norms and standards of host countries Availability and access for pre-kindergarten programs, open public spaces for children’s play, family literacy programs
Education and free time	Increased demand on schooling and education systems for boys and girls	Ensure developmental and intellectual stimulation for children at all stages of development along the migrant journey Improve child development and health monitoring for migrants Semi-structured active free time for play, visiting host-heritage sites (museums, galleries, concerts), organizing local tours, holidays, etc.
People with disabilities	Increased vulnerabilities along the migrant journey, including exposure to violence	Improve health monitoring of migrants with disabilities through improved record keeping Promote access to health, education, and employment services for those with disabilities
LGBT health	Increased vulnerability to depression, substance abuse, and acquiring HIV and other sexually transmitted infections	Eliminate discrimination and promote equal access to health services regardless of sexual orientation Provide education of risks and preventative interventions, both along the migrant journey and within the host country

Public health and its representatives have a duty to ensure the ethical guardianship of global health standards for all vulnerable groups—including migrants—through the use of their many professional competencies across sectors. Public health refers to all organized measures (whether public or private) to prevent disease, promote health, and prolong life among the population as a whole [10]. The WHO states that health is a state of complete physical, mental, and social well-being—not merely the absence of disease or infirmity [11]. Furthermore, health is a human right, based on the principles of justice, equity, and social solidarity. By transcending state boundaries to respond to public health challenges (such as the current migrant crisis in Europe), public health professionals and organizations can promote health for all and advancing well-being on a global scale.

History will judge how this crisis is addressed. The European community must draw from its collective memory of the massive displaced person experience following World War II and the Holocaust and from more recent recollections of the Balkan wars of the 1990s. Europe should be generous in giving humanitarian help for those who respect commonly accepted “European values” based on the principle of solidarity.

Sovereign states have concurrent concerns regarding the massive inflow of refugees, which may include security threats. In response, countries may introduce screening practices and may prefer legitimately documented refugees and survivors of genocidal action in their home countries to other migrants. Many countries will limit total migration to a number that can be managed and absorbed into the society while adhering to international law. We must work collectively (i) to avoid inconsistent practices and the introduction of new border restrictions and (ii) to ensure that international laws, ethical standards, and the rights of migrants and minorities are respected.

Solving the complex problems of migrant and minority health requires us to think through the interests and motivations of a number of actors, including governments, humanitarian agencies and their workers, academics, and the media. The organizational, financial, and human resource allocation needed to meet the health challenges of the current crisis will require high-level coordination at political, professional, and technical levels. Governments must work together with international governmental and non-governmental organizations to achieve consensus and share responsibilities and best practices on how to address migrant and minority health issues.

Addressing the health aspects of the migrant crisis is important because protecting and promoting migrant health is inextricably linked to public health. National governments have already demonstrated an ability to reach agreement on a number of areas concerning the current migrant crisis in Europe. The steps outlined in Table 1 for protection, basic needs, and health promoting activities are critical for addressing the health aspects of the migrant crisis facing Europe today.

## Appendix 1

**Table 2:** Salzburg Workshop on migrant and minority health, 3–9 April 2016 participants, rapporteurs, and working groups

Name	Affiliation/country	Contact
Organization		
1. Wolfgang Aulitzky	American Austrian Foundation	w.aulitzky@openmedicalinstitute.org
2. Laurent Chambaud	ASPHER/PHR Editor-in-chief/EHESP	laurent.chambaud@ehesp.fr
3. Jonathan Cohen	Open Society Foundations-NY	jonathan.cohen@opensocietyfoundations.org
4. Robert Otok	ASPHER	robert.otok@aspher.org
5. Sarabeth Harrelson	Open Society Foundations-NY	sarabeth.harrelson@opensocietyfoundations.org
Guest lecturers		
1. Isabel de la Mata	Principal Advisor Health and Crisis Management DG SANTE, European Commission	Isabel.delamata@ec.europa.eu
2. Santino Severoni	Coordinator Public Health and Migration WHO EURO	sev@euro.who.int
3. Thomas Nierle	President MSF, Switzerland	thomas.nierle@geneva.msf.org
4. Gustavo Fernandez	MSF, Switzerland	Gustavo.fernandez@geneva.msf.org
Faculty		
1. Dawn Davis	USA	ddavis32@slu.edu
2. Seth M. Holmes	USA/Germany	sethmholmes@berkeley.edu
3. Mark Johnson	UK	mrdj@dmu.ac.uk
4. Slava Plavinski	Russia	splavinskij@mail.ru
5. Bernd Rechel	UK	Bernd.Rechel@lshtm.ac.uk
6. Fredrik Saboonchi	Sweden	fredrik.saboonchi@rkh.se
7. Ted Tulchinsky	Israel	tulchinskyted@hotmail.com
8. Anita Villerusa	Latvia	Anita.Villerusa@rsu.lv
Fellows		
1. Kristina Astromske	Lithuania	astromske@gmail.com
2. Fabienne Azzedine	France	fabienne.azzedine@ehesp.fr
3. Lukasz Balwicki	Poland	balwicki@gumed.edu.pl
4. Levan Baramidze	Georgia	levan23@hotmail.com
5. Manana Beruchashvili	Georgia	mberuchashvili@yahoo.com
6. Tina Bregant	Slovenia	tina.bregant.drmed@gmail.com
7. Heide Castañeda	USA/Germany	hcastaneda@usf.edu
8. Nina Chala	Ukraine	ninachala@yandex.ua; chala_nina@ukr.net
9. Daniel Chemtob	Israel	daniel.chemtob@moh.health.gov.il
10. Alma Cicic	Montenegro	Alma.cicic@ijzcg.me
11. Catherine Cook	Canada	Catherine.Cook@umanitoba.ca
12. Jo Durham	Australia	m.durham@uq.edu.au
13. Ivan Froes	Ukraine	ifroes@iom.int, ipfroes@yahoo.com.br
14. Ana Hijaz	Spain	aihijas@fhalcorcon.es
15. Mariela Kamburova	Bulgaria	mariela_kamburova@yahoo.com
16. Gjuro Krenkovski	Macedonia	AAF/OMI fellow
17. George Koulierakis	Greece	gkoulierakis@esdy.edu.gr
18. Peter Krcho	Slovakia	peter.krcho@upjs.sk
19. Anna Kryukova	Russia	russia.omi@gmail.com
20. Raj Kumar Rai	India	rajesh.iips28@gmail.com
21. Vladimir Lazarevik	Macedonia	vlazarevik@healthgrouper.com
22. Oleg Lozan	Moldova	oleg.lozan@usmf.md
23. Bojana Matejic	Serbia	bmatejic@med.bg.ac.rs
24. Milena Santric-Milicevic	Serbia	msantric@med.bg.ac.rs
25. Lisa Ploeg	The Netherlands	l.ploeg@student.maastrichtuniversity.nl
26. Pawel Pludowski	Poland	p.pludowski@czd.pl
27. Lisa Rubin	Israel	lisa.rubin@MOH.GOV.IL
28. Amanda Shriwise	UK/USA	amanda.shriwise@spi.ox.ac.uk
29. Mariam Torosyan	Armenia	torosyan.mariam9@gmail.com
30. Dima Tsanova	Bulgaria	d_krumova@abv.bg
31. Christian Wejse	Denmark	wejse@dadlnet.dk
32. Elisabetta de Vito	Italy	devito@unicas.it
33. Hande Bahadir	Turkey	Handebahadir86@gmail.com
34. Stuart McClean	UK	Stuart.macclean@uwe.ac.uk
35. Marius Pop	Romania	mpop@iom.int

## Abbreviations

ASPHER: Association of Schools of Public Health in the European Region
IOM: International Organization for Migration
MSF: Médecins Sans Frontières
*PHR*: *Public Health Reviews*
WHO: World Health Organization

## Footnotes

1 Prof. Wolfgang Aulitzky (American Austrian Foundation), Laurent Chambaud (*PHR*), and Robert Otok (ASPHER) brought greetings from their respective organizations. Prof Ted Tulchinsky was the Workshop Coordinator, Mariam Torosyan was the Rapporteur, and Lukasz Balwicki was the Working Group Coordinator. Tina Bregant prepared a summary of the Workshop, and Amanda Shriwise assisted in preparing and editing the report.
